# Single-molecule conductance of dipyridines binding to Ag electrodes measured by electrochemical scanning tunneling microscopy break junction

**DOI:** 10.1186/1556-276X-9-77

**Published:** 2014-02-17

**Authors:** Xiao-Yi Zhou, Ya-Hao Wang, Han-Mei Qi, Ju-Fang Zheng, Zhen-Jiang Niu, Xiao-Shun Zhou

**Affiliations:** 1 Zhejiang Key Laboratory for Reactive Chemistry on Solid Surfaces, Institute of Physical Chemistry, Zhejiang Normal University, Jinhua, Zhejiang 321004, China

**Keywords:** Sliver, Single-molecule junctions, STM-BJ, Charge transport, Electrochemistry

## Abstract

We have measured the conductance of three pyridyl-terminated molecules binding to Ag electrodes by using electrochemical jump-to-contact scanning tunneling microscopy break junction approach (ECSTM-BJ). Three molecules, including 4,4′-bipyridine (BPY), 1,2-di(pyridin-4-yl)ethene (BPY-EE), and 1,2-di(pyridin-4-yl)ethane (BPY-EA), contacting with Ag electrodes show three sets of conductance values, which follow the order of BPY > BPY-EE > BPY-EA. These values are smaller than those of molecules with Au electrodes, but larger than those of molecules with Cu electrodes. The difference may attribute to the different electronic coupling efficiencies between the molecules and electrodes. Moreover, the influence of the electrochemical potential on the Fermi level of electrodes is also discussed.

## Background

Single metal-molecule-metal junctions have attracted much attention for their fundamentally important role in molecular electronics [1-3]. While the molecular structure is demonstrated to influence the charge transport through single-molecule conductance [4,5], the contact between electrode and molecule also plays an important role [6,7]. For example, the electrode materials can influence the electronic coupling between electrodes and molecules, such as the interaction of electrode-anchoring group and the alignment of the energy level of electrode-molecule [8,9]. Typically, most of the conductance measurements of single-molecule junctions were performed by using Au as electrode for its chemically inert property [10]. However, it is also important to study the non-Au electrodes to fully understand the charge transport through single-molecule junctions. We pay attention to the Ag electrodes for the following reasons: Ag has strong optical enhancement property and high catalytic activity [10-12]. It has a similar electronic structure with Au and Cu and is easy for comparison among them.

Single-molecule conductance can be measured by scanning tunneling microscopy (STM) break junction (STM-BJ), mechanically controllable break junction (MCBJ), STM trapping and conducting atomic force microscopy, and so on [13-21]. Though lots of works have been done on the electron transport of single-molecule junctions by using the above methods, there is limited investigation on single-molecule junctions with non-Au electrodes [10,22].

**Figure 1:**
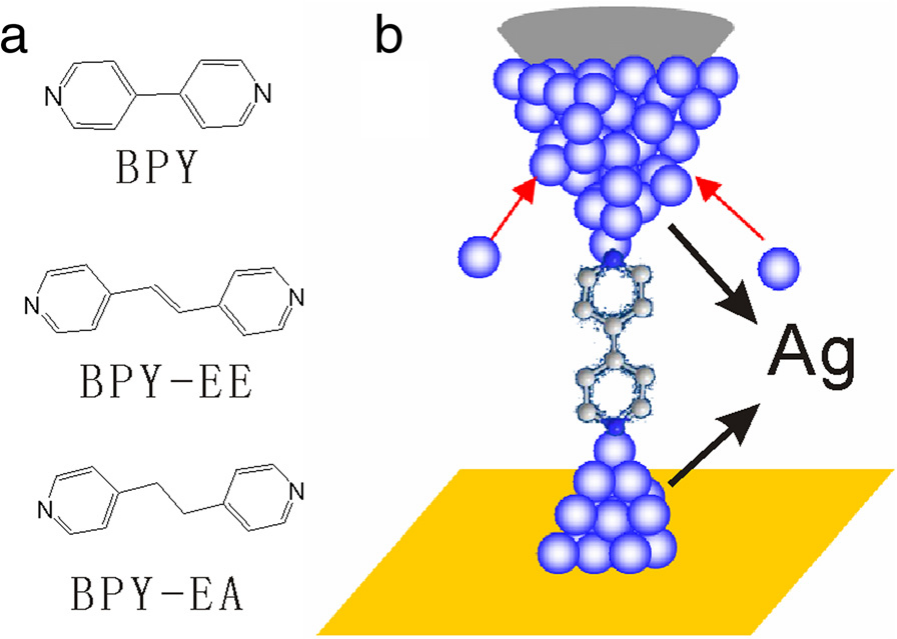
**Molecular structure and schematic diagram of ECSTM-BJ. (a)** Molecular structures of 4,4′-bipyridine (BPY), 1,2-di-(pyridin-4-yl)ethene (BPY-EE), and 1,2-di(pyridin-4-yl)ethane (BPY-EA), and **(b)** schematic diagram of Ag-molecule-Ag junctions formed by the ECSTM-BJ.

We have developed an electrochemical jump-to-contact scanning tunneling microscopy break junction approach (ECSTM-BJ) [23]. By using this approach, single-molecule junctions with carboxylic acid binding to different metallic electrodes were systematically investigated [9,24]. Since the pyridyl group also has received much attention [15,17,25-27], we recently extended this approach to the conductance measurement of pyridyl-based molecules binding to Cu electrode, which shows that the single-molecule conductance with pyridyl-Cu contacts is smaller than that with pyridyl-Au contacts [28]. In this work, we focus on the single-molecule junctions with pyridyl group (Figure 1a) binding to Ag contacts by ECSTM-BJ. Especially, the influence of the electrochemical potential on the Fermi level of electrode is discussed.

## Methods

Au(111) was used as substrate, and mechanically cut Pt-Ir (*Φ* = 0.25 mm) wires were used as the tips. The latter was insulated by the thermosetting polyethylene glue to reduce the leakage current of the electrochemical reaction. Ag and Pt wire were used as the reference and counter electrodes, respectively. 1,2-Di(pyridin-4-yl)ethene (BPY-EE) and 1,2-di(pyridin-4-yl)ethane (BPY-EA) were purchased from Sigma-Aldrich Corp. (St. Louis, MO, USA), while 4,4′-bipyridine (BPY) and Ag_2_SO_4_ (99.999%) were purchased from Alfa Aesar (Ward Hill, MA, USA). H_2_SO_4_ was purchased from Sinopharm Chemical Reagent Co., Ltd. (Shanghai, China). All aqueous solutions were prepared with ultrapure water (>18 MΩ cm).

The conductance of the Ag-molecule-Ag junctions was measured by repeatedly forming and breaking the molecular junctions on the modified Nanoscope IIIa STM (Veeco Instruments, Inc., Plainview, NY, USA), and the process was described in detail in our previously reports (Figure 1b) [9,28]. To achieve this process, Ag was continuously electrodeposited onto the STM tip. Then, the deposited tip was pulled far away from the substrate about several tens of nanometers with the STM feedback disabled. Next, the tip was driven towards the surface until a certain tip current was reached; the atoms of the deposited metal on the tip would transfer to the substrate upon the application of a pulse on the *z*-piezo of STM, and this is the so-called jump-to-contact process. Atomic-sized wire of the deposited metal could be obtained by pulling the tip out of the contact. Lastly, the molecular junctions with the deposited metal as electrode were formed after breaking of the atomic-sized metal wire. Conductance curves were recorded at the same time. Then, we moved the tip to other positions and repeated the whole process. Typically, large conductance traces were obtained, and hundreds from thousands traces with clear stepwise features were selected to get a statistical result. The selection rate is around 15%, which is similar as that of pyridyl-Cu contact in an acidic solution in our previously report [28]. The low selection rate may be caused by the protonated pyridyl group [28]. All experiments were carried out at a fixed bias voltage of 50 mV.

## Results and discussion

### Conductance of BPY-EE contacting with Ag electrodes

The conductance of Ag-(BPY-EE)-Ag junctions was measured in 0.05 M H_2_SO_4_ aqueous solution containing 1 mM Ag_2_SO_4_ and 0.5 mM BPY-EE by using the ECSTM-BJ approach. In order to avoid the deposition of Ag^+^ and pyridyl group in a neutral solution, the acidic supporting electrolyte was used. Though the pyridyl group is in protonated form in this acidic solution, it may contact with the electrode through a deprotonated form [28]. The Au(111) substrate and Pt-Ir tip were set at 45 and −5 mV vs the Ag wire, respectively.

**Figure 2:**
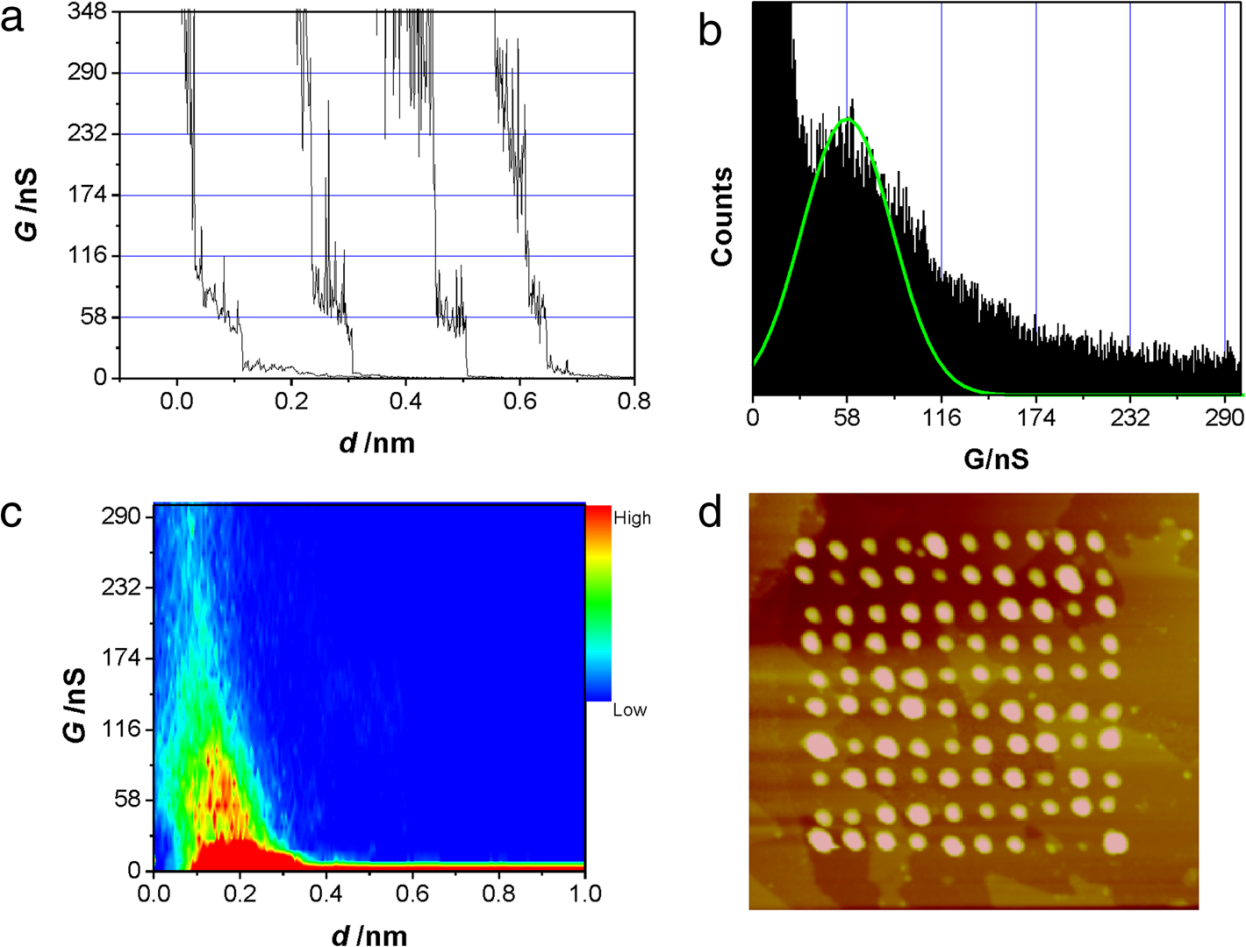
**High conductance of the Ag-(BPY-EE)-Ag junctions. (a)** Typical conductance curves for high conductance (HC) of Ag-(BPY-EE)-Ag junctions. **(b)** 1D and **(c)** 2D conductance histogram of the Ag-(BPY-EE)-Ag junctions constructed from the curves shown in **(a)**. **(d)** The STM image (150 × 150 nm^2^) of a 10 × 10 array of Ag clusters simultaneously generated with the conductance curves.

**Figure 3:**
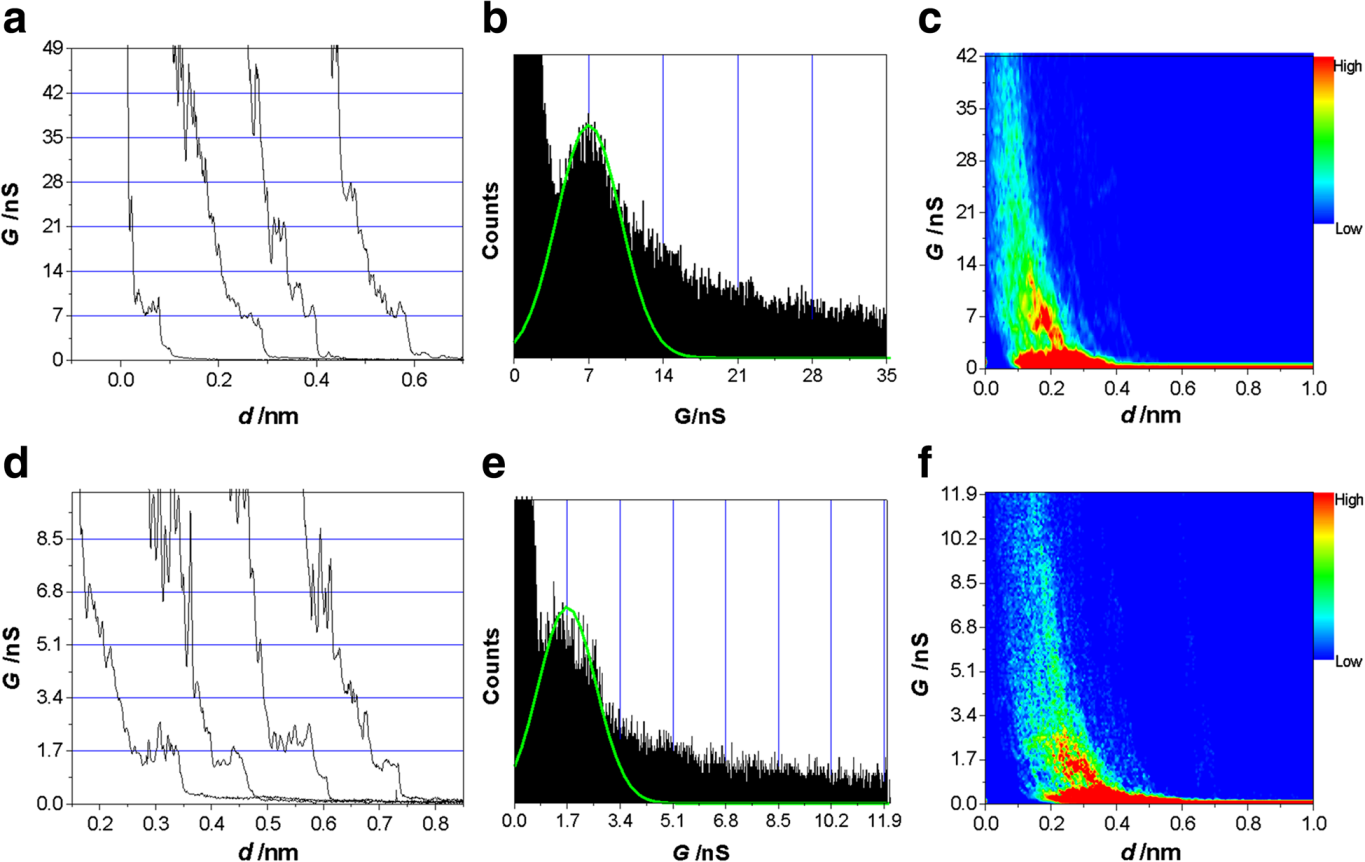
**Medium and low conductance of the Ag-(BPY-EE)-Ag junctions.** Typical conductance curves for **(a)** medium conductance (MC) and **(d)** low conductance (LC) of the Ag-(BPY-EE)-Ag junctions. **(b)** MC and **(e)** LC of 1D conductance histogram of single-molecule junctions of Ag-(BPY-EE)-Ag. **(c)** MC and **(f)** LC of 2D conductance histograms of single-molecule junctions of Ag-(BPY-EE)-Ag.

Figure 2a shows the typical conductance curves of Ag-(BPY-EE)-Ag, presenting a rapid drop from step of 58 ± 32 nS ((7.5 ± 4.2) × 10^−4^*G*_0_). The one-dimensional conductance histogram constructed from hundreds of such individual conductance traces reveals single-molecule conductance values of 58 ± 32 nS (Figure 2b), and the conductance value is the same as that of a two-dimensional (2D) histogram (Figure 2c), which is constructed by counting the number of data at each conductance value with each stretching distance from the conductance curves [9,29]. In other words, individual data points are binned in a two-dimensional histogram (the bin size for the distance is 0.005 nm), while the conductance value for the (BPY-EE)-Ag contact in Figure 2c is 8.9 nS (0.89 nS for Figure 3c and 0.056 nS for Figure 3f). Typically, a conductance value close to a saturated range of the scanner amplifier was set for zero distance in the 2D histograms. The high counts can represent the most typical breaking behavior of the molecular junctions in such 2D histogram. We can also get the 10 × 10 arrays of the Ag clusters, which were formed simultaneously by the breaking of the junctions as shown in Figure 2d.

Two more sets of conductance values 7.0 ± 3.5 nS ((0.90 ± 0.46) × 10^−4^*G*_0_) (Figure 3a,b,c) and 1.7 ± 1.1 nS ((0.22 ± 0.14) × 10^−4^*G*_0_) (Figure 3d,e,f) were also found for the Ag-(BPY-EE)-Ag junctions. These are consistent with the contacts with Cu and Au, which also have three sets of conductance values [17,27,28]. The multiple conductance values can be contributed to the different contact configurations between the electrode and anchoring group [7,30]. The conductance values 58 ± 32, 7.0 ± 3.5, and 1.7 ± 1.1 nS can be denoted as high conductance (HC), medium conductance (MC), and low conductance (LC), respectively. Taking the HC value as example, the conductance values for pyridyl-Cu and pyridyl-Au are 45 and 165 nS, respectively, as reported by our group [28]. The conductance value of pyridyl-Ag is in between them. Moreover, it also shows the same order for the MC and LC with different metal electrodes. The different conductance values can be contributed to the different electronic coupling efficiencies between the molecules and electrodes [9]. We will discuss it later.

### Conductance of BPY and BPY-EA contacting with Ag electrodes

**Figure 4:**
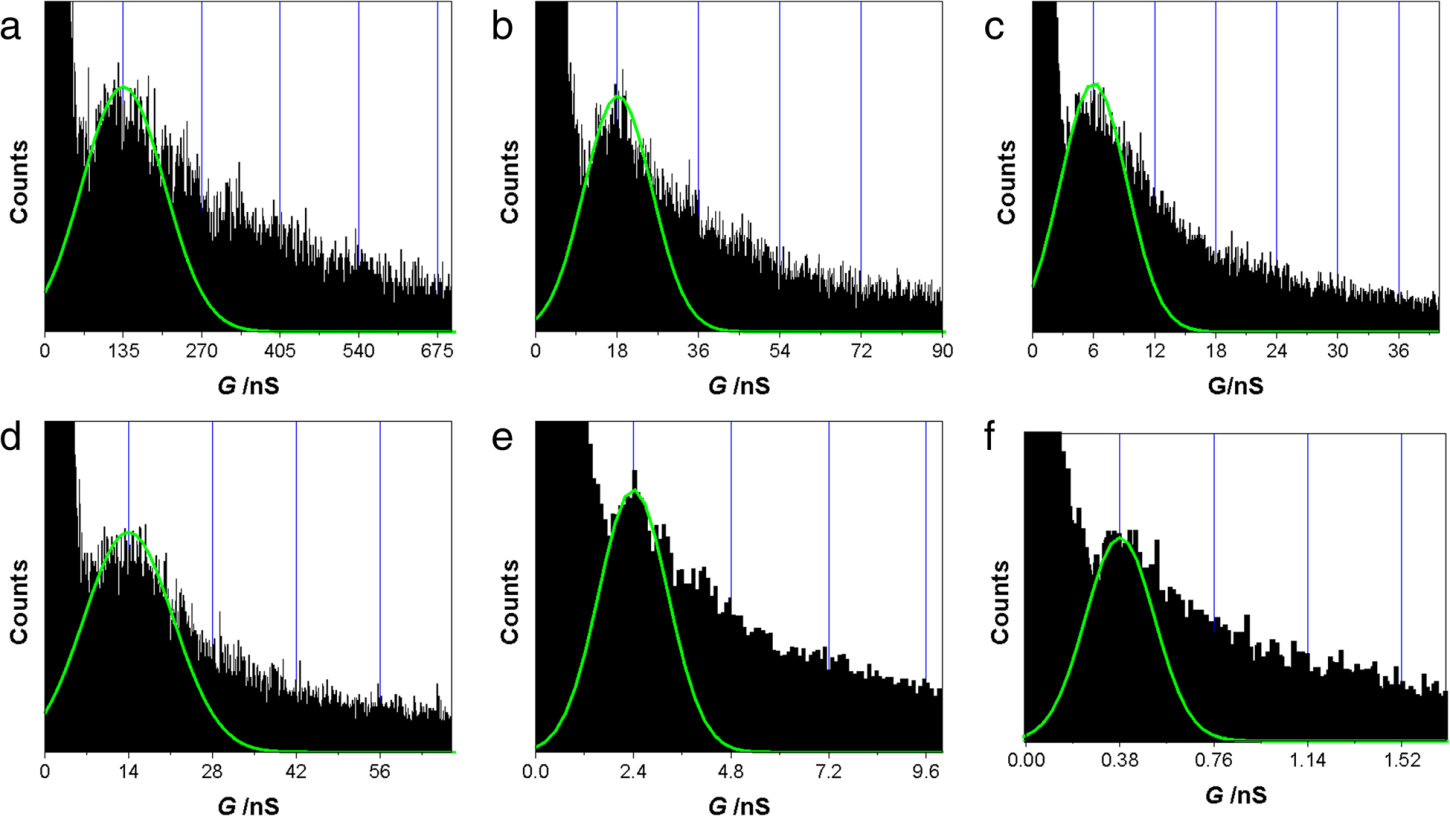
**HC, MC, and LC of the Ag-BPY-Ag junctions. (a)** HC, **(b)** MC, and **(c)** LC histograms of the BPY junctions contacting with Ag. **(d)** HC, **(e)** MC, and **(f)** LC histograms of the BPY-EA junctions contacting with Ag.

We also carried out the conductance measurement of BPY and BPY-EA contacting with Ag electrodes by using the same method. The results are shown in Figure 4. The HC, MC, and LC of BPY are 140 ± 83 nS ((18.1 ± 10.7) × 10^−4^*G*_0_), 19.0 ± 8.8 nS ((2.4 ± 1.1) × 10^−4^*G*_0_), and 6.0 ± 3.8 nS ((0.78 ± 0.49) × 10^−4^*G*_0_), while those of BPY-EA are 14.0 ± 8.8 nS ((1.8 ± 1.1) × 10^−4^*G*_0_), 2.4 ± 1.1 nS ((0.31 ± 0.14) × 10^−4^*G*_0_), and 0.38 ± 0.16 nS ((0.049 ± 0.021) × 10^−4^*G*_0_), respectively. The single-molecule conductance values of BPY, BPY-EE, and BPY-EA are summarized in Table 1.

**Table 1:** Summary of single-molecule conductance with contact of the Ag electrodes

**Molecules**	**HC (nS)**	**MC (nS)**	**LC (nS)**
BPY	140 ± 83	19.0 ± 8.8	6.0 ± 3.8
BPY-EE	58 ± 32	7.0 ± 3.5	1.7 ± 1.1
BPY-EA	14.0 ± 8.8	2.4 ± 1.1	0.38 ± 0.16

Taking the HCs of BPY (140 ± 83 nS), BPY-EE (58 ± 32 nS), and BPY-EA (14.0 ± 8.8 nS) as examples, the conductance of BPY is about twice that of BPY-EE, and 10 times that of BPY-EA. Though BPY-EE and BPY-EA have similar lengths of 0.95 nm, BPY-EE is kept with conjugated backbone, while the conjugated backbone is interrupted by the insertion of CH_2_CH_2_ in BPY-EA [25,31]. These facts have contributed to the big difference between the conductance of BPY-EE and BPY-EA. The conductance values of BPY and BPY-EA contacting with Ag are also in between those of BPY and BPY-EA contacting with Au and Cu electrodes.

### The influence of the metal electrodes on the single-molecule conductance

**Figure 5:**
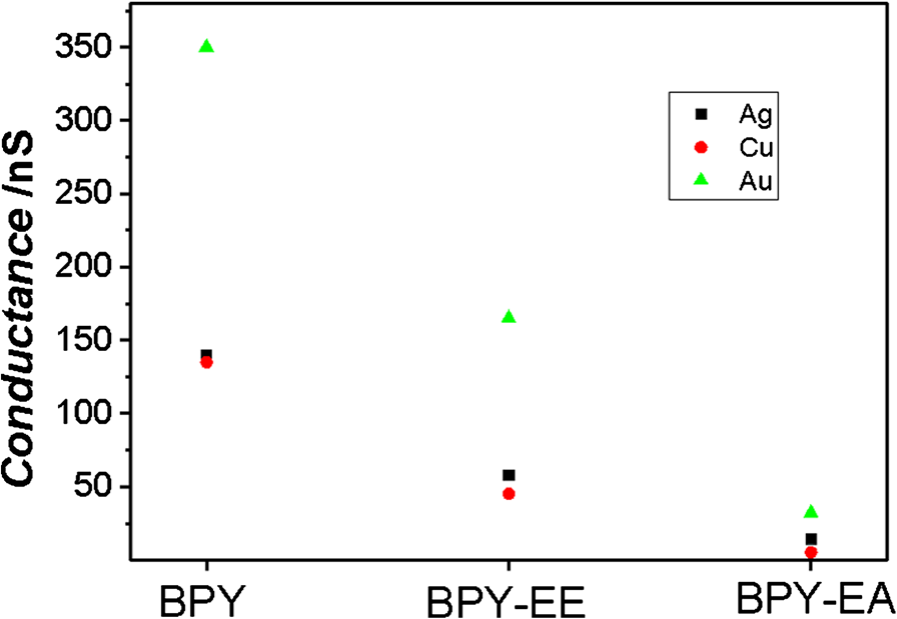
**HC of BPY, BPY-EE, and BPY-EA contacting with Ag, Cu, and Au electrodes.** HC of single-molecule junctions of BPY, BPY-EE, and BPY-EA contacting with Ag, Cu, and Au electrodes. The data for Cu and Au are from Zhou et al. [28].

Now, we will focus on the influence of metal electrodes on the single-molecule conductance. We compare the single-molecule conductance contacting with Ag, Au, and Cu electrodes. Taking the HC as example, the conductance value of pyridyl-Ag is between the values of pyridyl-Au and pyridyl-Cu as shown in Figure 5. It is in the same order for the MC and LC with different metal electrodes. It was reported that the binding interaction of pyridyl with Ag, Cu, and Au follows the order of pyridyl-Cu ~ pyridyl-Au > pyridyl-Ag by theoretical calculation [32], which is different from the conductance value order of pyridyl-Au > pyridyl-Ag > pyridyl-Cu. Thus, the conductance difference may mainly be contributed to the efficiency of electron transport along the molecule for Cu, Au, and Ag [28].

It was reported that the LUMO is the essential orbital channel for the electron transport in the Au-BPY-Au junction without potential control of the electrodes [26,27]. However, the situation may be complex in the current experiment with the control of the electrode potential. The Fermi level of the electrode would be changed by the potential. Usually, the Fermi energy of the hydrogen reference electrode under standard conditions (SHE) is considered as the zero energy in electrochemistry, while the energy of SHE is very close to 4.44 eV [33]. Typically, the standard potentials for the Ag^+^|Ag and Cu^2+^|Cu are 0.80 V (SHE) and 0.34 V (SHE), respectively [34]. If we consider the influence of the concentrations of the metal ion (1 mM Ag_2_SO_4_ and 1 mM CuSO_4_), the potentials for the equilibria

$$Ag^{+}\;\left(1\mathrm{mM}\right)+e^{-}↔\mathrm{Ag}$$

$$Cu^{2+}\left(1\mathrm{mM}\right)+2e^{-}↔\mathrm{Cu}$$

are 0.64 V (SHE) and 0.25 V (SHE), respectively. We also measured the potentials of the Ag^+^|Ag in the aqueous solution containing 0.05 M H_2_SO_4_ + 1 mM Ag_2_SO_4_ + 0.5 mM BPY and Cu^2+^|Cu in the 0.05 M H_2_SO_4_ + 1 mM CuSO_4_ + 0.5 mM BPY, which give out the 0.65 V (SHE) for Ag^+^|Ag and 0.25 V (SHE) for Cu^2+^|Cu. Correspondingly, these values are similar with the above calculated values. We can infer that the Fermi energy levels for Ag^+^|Ag and Cu^2+^|Cu are −5.09 and −4.69 eV from the measured potentials, respectively. For the Au electrode, we found that the potential of Au wire is about 0.45 V in 50 mM H_2_SO_4_ + 0.5 mM BPY and give out −4.89 eV for the Fermi energy of Au. Returning back to our experiments, the electrodes were controlled near the potentials of the reference wires (Ag, Cu, and Au) [28]; thus the Fermi energy of the electrode may also be approximated to these energy levels. However, these values are quite different from the Fermi energy of Au (−5.13 eV), Ag (−4.65 eV), and Cu (−4.26 eV) in vacuum [35], and may change the essential orbital channel of the molecules.

It is not possible to know which orbital channel (such as HOMO or LUMO) is actually the most favorable in the current study. However, the conductance order of the single-molecule junctions with different metallic electrodes is caused by the different coupling efficiency between the metallic electrodes and the anchoring group, and also the molecular energy levels and Fermi energy level of the electrodes [8,9]. Further calculations are needed to fully understand the influence of the metallic electrodes.

## Conclusions

We have measured the single-molecule conductance of pyridine-terminated molecules contacting with Ag electrodes. All three molecules (BPY, BPY-EE, and BPY-EA) have three sets of conductance values and show the order of BPY > BPY-EE > BPY-EA. These values are larger than those of molecules with the Cu electrodes, but smaller than those of molecules with the Au electrodes. The different single-molecule conductance between Ag, Cu, and Au electrodes can be attributed to the different electronic coupling efficiencies between the molecules and electrodes.

## Competing interests

The authors declare that they have no competing interests.

## Authors’ contributions

XYZ and YHW carried out the experiments. HMQ analyzed the results. XSZ, XYZ, JFZ, and ZJN conceived and designed the experiments, analyzed the results, and wrote the manuscript. All authors read and approved the final manuscript.

## Authors’ information

XYZ is a Master's degree student under the supervision of XSZ in the Institute of Physical Chemistry, Zhejiang Normal University, China.
